# Ghanaian women’s experience of intimate partner violence (IPV) during group antenatal care: a brief report from a cluster randomised controlled trial

**DOI:** 10.1080/16549716.2024.2325250

**Published:** 2024-04-05

**Authors:** Samia J. Abdelnabi, Michelle L. Munro-Kramer, Cheryl A. Moyer, John E.O. Williams, Jody R. Lori

**Affiliations:** a School of Nursing, University of Michigan, Ann Arbor, MI, USA; b Learning Health Sciences, University of Michigan, Ann Arbor, MI, USA; c Dodowa Health Research Centre, Ghana Health Service, Dodowa, Ghana

**Keywords:** Violence, Africa, Ghana, pregnancy, self-efficacy, safety, education

## Abstract

Intimate partner violence (IPV) impacts women of reproductive age globally and can lead to significant negative consequences during pregnancy. This study describes an exploratory aim of a cluster randomised controlled trial designed to assess the outcomes of Group Antenatal Care (ANC) in Ghana. The purpose was to understand the effect of a healthy relationship Group ANC module on experiences of IPV and safety planning as well as to explore the relationship between self-efficacy on the experiences of IPV and safety planning. Data were collected at baseline and at 11–14 months postpartum (post). Survey measures captured reported experiences of violence, self-efficacy, and safety. The chi-square test was used to compare baseline and post scores, and a logistic regression was performed to ascertain the effects of self-efficacy on the experiences of IPV in both groups. The sample included 1,751 participants, of whom 27.9% reported IPV at baseline. Between baseline and postpartum, there was a small increase in reported emotional (6.2% vs. 4.6%) and sexual (5.4% vs. 3.2%) violence in the intervention group compared to the control group. Logistic regression demonstrated that an increasing self-efficacy score was associated with an increased likelihood of experiencing IPV. There were no changes in safety knowledge. This study found higher rates of reported sexual and emotional violence post-intervention among the intervention group. Group ANC may be just one part of a portfolio of interventions needed to address IPV at all socio-ecological levels.

**Paper Context**
- **Main findings:** There was no reduction in experiences of intimate partner violence or increases in safety planning among Ghanaian pregnant women participating in a Group Antenatal Care session focused on healthy relationships and safety planning.
- **Added knowledge:** Group Antenatal Care has been identified as an effective modality for providing antenatal care and facilitating conversations about sensitive topics such as intimate partner violence and safety. However, this study highlights the importance of developing multifaceted approaches to decrease the risk of intimate partner violence among women, especially during the critical times of pregnancy and postpartum.
- **Global health impact for policy and action:** Effective global health action and policy must extend beyond educational efforts, incorporating multifaceted strategies that include healthcare provider training, robust community engagement, and legislation aimed at preventing intimate partner violence, with a special focus on safeguarding the well-being of women during pregnancy and the postpartum period.

## Introduction

Sexual and physical violence by an intimate partner is estimated to impact 27% of women globally [1]. In Africa, intimate partner violence (IPV) is experienced by 57% of women, resulting in negative physical and psychological consequences [2]. Alangea and colleagues explored rates of IPV among a population-based sample of 2000 reproductive-age women and found that the rates over the last year were 24.6% for emotional violence, 15.5% for physical violence, and 11.8% for sexual violence [2]. During the reproductive age, particularly during pregnancy, IPV can lead to detrimental outcomes for both the mother and the foetus, including gestational hypertension, low birth weight, foetal distress, and maternal death [3]. Researchers have identified IPV as a barrier in accessing and utilising antenatal care (ANC) [4]. Pregnant women in sub-Saharan Africa who experienced IPV during pregnancy were less likely to utilise timely ANC [4].

A feasibility trial of Group ANC in Ghana suggested that it might improve the experiences of pregnant women and providers [5,6]. It also provides the opportunity to deliver education in a group setting, which has been touted by survivors as a desirable way to learn about IPV [7]. This study describes an exploratory aim of a cluster randomised controlled trial (RCT) designed to assess the outcomes of Group ANC in Ghana [8]. Specifically, the purpose of this exploratory aim was to understand the effect of a healthy relationship Group ANC module on the experiences of IPV and safety planning. We hypothesise that those in Group ANC will report fewer experiences of IPV in the postpartum period and demonstrate higher scores in safety planning. Additionally, we aimed to explore the relationship between self-efficacy on experiences of IPV and safety planning.

## Methods

### Parent study

The **Gr**oup **An**tenatal Care **D**elivery Project (GRAND) is a cluster RCT (NCT04033003) designed to improve health literacy, increase birth-preparedness and complication readiness, and optimise maternal and newborn outcomes among women attending Group ANC in the eastern region of Ghana [8]. Fourteen facilities were randomised using a matched-pair method, where one site was randomly assigned to Group ANC (intervention) and the other to routine individual ANC (control). The Group ANC intervention took place in seven health facilities, with anywhere from 10 to 14 pregnant women grouped together by gestational age at the first ANC visit. Women continued to meet with the same midwife throughout the pregnancy, receiving gestationally appropriate education through a facilitated discussion using storytelling, picture cards, role-play, and demonstrations [8]. The control group included seven health facilities that received usual care (e.g. one-on-one visits with the midwife) [8]. The study was approved by the University of Michigan Health Sciences Behavioral Sciences Institutional Review Board (HUM00161464) and Ghana Health Service Ethics Review Committee (GHS-ERC016/04/19), and informed consent was obtained in the participants’ language prior to study participation.

### Healthy relationship intervention

**Figure 1.**
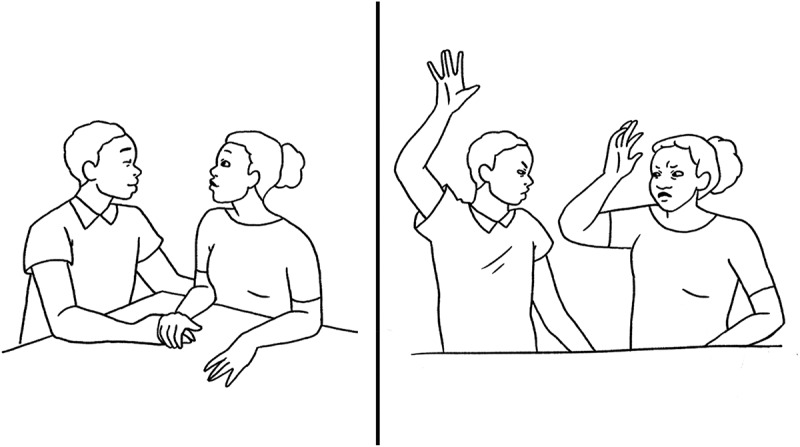
Picture card of verbal/emotional abuse shown to participants.

For this exploratory aim, two case studies and a picture card (see Figure 1) were used at 16–20 weeks’ gestation to facilitate a discussion around conflict management and safety strategies. The two cases focused specifically on 1) emotional and physical abuse, including a facilitated discussion on the acceptability of violence and actions that a woman experiencing such violence can take, and 2) communication and safety tips for individuals experiencing violence within the Ghanaian context, including a list of formal (e.g. police and healthcare providers) and informal resources (e.g. family and friends).

Experiences of physical, sexual, and emotional violence were measured using four items from the WHO measures on IPV [1]. Self-efficacy was measured with a Jhpiego-developed scale [9] and analysed as a composite score: low (0–3), moderate, (4–6), and high (7–8). Safety was measured with four investigator-derived questions about the participant’s comfort in finding a safe place (e.g. I can find a safe place when my husband/partner is acting violently) or seeking out resources when experiencing IPV (e.g. I know some tactics to calm my husband/partner down when he is yelling at me) using dichotomous responses (composite score range: 0–4). Data for this exploratory aim were collected at baseline (<20 weeks’ gestational age) using an in-person survey administered by a research assistant and at 11–14 months postpartum (post) using a structured phone interview.

### Analyses

All analyses were conducted using Stata version 17.0 (StataCorp LLC). The chi-square test was used to compare baseline and post scores, and a logistic regression was performed to ascertain the effects of age, education, and self-efficacy on the experience of IPV in both groups. Mediating analysis was performed by assessing whether and the extent to which the intervention effect (i.e. difference in IPV outcome between control and treatment) changed before and after controlling for safety (have a safety plan or are aware of a safety net). Moderator analysis was then completed using a logistic model to determine whether there was an interaction effect between the intervention and baseline self-efficacy (i.e. significant variation in the intervention effect depending on baseline self-efficacy categories).

## Results

The mean age of women in the control and intervention groups was 28 years (range: 15–51 years). Education level was similar in both groups, with at least half of the participants completing middle school. Most participants identified as Christian and were in some type of relationship. See Table 1 for additional demographic characteristics.

**Table 1.** Demographic data of intervention and control population.

		Overall	Control	Intervention	
		*N* = 1761	*N* = 884	*N* = 877	*p*-value
Age					
	Less than 25	501 (28%)	266 (53%)	235 (47%)	0.193
	25–34	987 (56%)	477 (48%)	510 (52%)	
	35 or more	273 (16%)	141 (52%)	132 (48%)	
Relationship					
	Single/Divorced/Widowed	70 (4%)	53 (76%)	17 (24%)	<0.0001
	Married/Cohabitating/Living Together	1691 (96%)	831 (49%)	860 (51%)	
Maternal Education					
	Primary	246 (14%)	120 (49%)	126 (51%)	0.6895
	Middle/JHS/JSS	829 (49%)	429 (52%)	400 (48%)	
	Secondary/SHS/Technical/Vocational	459 (27%)	223 (49%)	236 (51%)	
	Tertiary	164 (10%)	83 (51%)	81 (49%)	
Partner Education					
	Middle/JHS/JSS or Less	666 (39%)	335 (50%)	331 (50%)	0.8525
	Secondary	627 (37%)	306 (49%)	321 (51%)	
	Tertiary	261 (16%)	126 (48%)	135 (52%)	
	N/A, Unknown	137 (8%)	64 (47%)	73 (53%)	
Religion					
	Christianity	1646 (93%)	835 (50.4%)	811 (49.6%)	0.1185
	Muslim	97 (6%)	39 (39%)	58 (61%)	
	Other	18 (1%)	10 (56%)	8 (44%)	
First pregnancy					
	No	1412 (80%)	703 (50%)	709 (50%)	0.4876
	Yes	349 (20%)	181 (52%)	168 (48%)	
Location of Delivery					
	Hospital/Polyclinic/Health Centre	1711 (97%)	853 (50%)	858 (50%)	0.0904
	Other	50 (3%)	31 (62%)	19 (38%)	

A total of 1761 women participated in the study, and the final sample size for this analysis was 1751. At baseline, the prevalence of IPV among all participants was 27.9%; there were no differences in rates of physical, sexual, or emotional violence between groups at baseline. Between baseline and one-year postpartum, there was a small increase in changes in reported emotional violence (from no to yes) in the intervention group compared to the control group (6.2% vs. 4.6%) and sexual violence in the intervention group compared to the control group (5.4% vs. 3.2%). See Table 2 for additional details on changes from baseline to post-intervention.

**Table 2.** Change in IPV^a^ self-reports from baseline to post-intervention.

	Post (*n* = 1145)		
Types of IPV	Intervention (*n* = 553)	Control (*n* = 592)	*p*-value
Physical	2 (0.36%)^a^	13 (2.20%)^a^	*p* = 0.019
Low Self-Efficacy	0 (0.00%)	0 (0.00%)	*p* = 0.305
Moderate Self-Efficacy	1 (0.72%)	4 (2.08%)	*p* = 0.260
High Self-Efficacy	1 (0.26%)	9 (2.43%)	*p* = 0.033
Sexual	30 (5.43%)	19 (3.20%)	*p* = 0.052
Low Self-Efficacy	0 (0.00%)	1 (3.45%)	*p* = 0.080
Moderate Self-Efficacy	10 (7.25%)	4 (2.07%)	*p* = 0.020
High Self-Efficacy	20 (5.22%)	14 (3.76%)	*p* = 0.375
Emotional	34 (6.17%)	27 (4.57%)	*p* = 0.349
Low Self-Efficacy	2 (6.67%)	1 (3.45%)	*p* = 0.318
Moderate Self-Efficacy	12 (8.70%)	6 (3.148%)	*p* = 0.090
High Self-Efficacy	20 (5.39%)	20 (5.39%)	*p* = 0.925

^a^Number of women who denied experiencing violence at baseline and then reported experiencing violence at follow-up.

Logistic regression demonstrated that age and education were not associated with reports of IPV. There were no changes in safety knowledge among the intervention group from baseline to post-intervention (R2 = 0.00, F(1, 1155) = 1.36, *p* = 0.244, 95% CI [−0.21, 0.055]). We found no mediating effect (i.e. no change in OR before and after adding safety). We also did not see a moderating effect. More specifically, there was a significant adverse intervention effect (*p* = 0.002, 95% CI [0.339, 0.1543]) for those in moderate self-efficacy group (score of 4–6), but there was no significant intervention effect for the other self-efficacy groups (low: *p* = 0.95 [−0.1295, 0.1382]; high: *p* = 0.71 [−0.2385, 0.0507]). However, the significant adverse effect was not statistically different from other groups with a null intervention effect.

## Discussion

The prevalence rate of IPV in our sample (27.9%) is consistent with previous literature that has found that 24–27% of Ghanaian women reported experiencing IPV [2,10]. After the Group ANC educational intervention on IPV, we did not find a decrease in women reporting IPV between the first trimester of pregnancy and the postpartum period. Rather, we noticed higher reported rates of sexual and emotional violence in the postpartum period among participants in the Group ANC intervention. This could be related to elevated rates of IPV that have been reported among Ghanaian women during pregnancy [3], or it could indicate a better understanding by the women enrolled in Group ANC of the different and often less recognised forms of IPV (e.g. sexual and emotional). Owusu Adijah and Agbemafle [11] found that sexual violence was the least reported form of IPV by Ghanaian women, which could indicate a lack of recognition of sexual violence as a form of IPV.

Another consideration for the elevated rates of sexual and emotional IPV reported in the postpartum period may also be related to data collection methods, which changed from in-person at baseline to over the phone during the postpartum period. Research on clinical screening for IPV has demonstrated that patients are more likely to report experiences of violence using technology than through face-to-face screening [12].

The stigma of IPV could also be a barrier to disclosure, as women who disclose experiences of IPV to formal or informal support networks have encountered negative reactions to their disclosure that may be culturally mediated [4,13]. For example, Rodríguez and colleagues [14] found that lack of utilisation of healthcare services by ethnically diverse IPV survivors was attributed to worries about stigmatisation, beliefs that IPV should not be discussed with others, and cultural stigmatisation around seeking out mental health care.

Another finding in this study is that moderate self-efficacy increased women’s reporting of IPV. This could be due to the level of acceptance of IPV. Women who are educated are more likely to have moderate-to-high self-efficacy compared to those with low self-efficacy; thus, they are less likely to accept IPV. A study by Adu [15] found that women with no education (47.3%) were more likely to justify IPV compared to those with higher education (3.6%).

Finally, safety knowledge did not change post-intervention. This could be due to the lack of resources available in Ghana or a lack of awareness about what is available. According to Anyemedu, Tenkorang, and Dold [16], Ghanaian women stated that they had heard of the Domestic Violence and Victim Support Unit of the Ghana Police Service but lacked knowledge on the types of support provided. They also expressed doubt that these services could adequately handle cases of IPV.

This study was limited by a focus on one geographic region in Ghana, with limited formal help-seeking resources. Also, the intervention only examined an educational model on experiences of IPV and safety planning outcomes at the individual level rather than at other levels such as within the family or community. The healthcare providers running the Group ANC intervention were trained in group facilitation techniques but did not receive training on screening for or discussing IPV.

Overall, help-seeking among women exposed to IPV in sub-Saharan Africa is low at 38.8%; thus, interventions must address the full range of socio-ecological factors (e.g. individual-level knowledge and attitudes, relationship-level factors, community-level factors, and societal-level factors) [4]. In Ghana, there are limited formal help-seeking supports across the country, such as shelters and social welfare offices. Current attempts to provide these services have been regionally specific (e.g. the construction of a shelter in Accra and the monthly Abuse Clinic in Kumasi). These limited structural interventions may not provide the infrastructure needed for survivors, or adequate resources that can be recommended by healthcare providers. Additionally, many healthcare providers have limited training in screening for and managing IPV, which could lead to hesitation about reporting or seeking assistance [17,18]. There are scholars and clinicians evaluating interventions for IPV in sub-Saharan Africa [19]; however, additional work is needed to explore how these interventions impact sub-populations such as pregnant women.

## Conclusions

Safety planning has been seen as a viable option for support in other low- and middle-income countries with limited formal help-seeking resources [20] and should continue to be considered as part of a more comprehensive approach to address IPV among pregnant women in Ghana. Group ANC may be just one part of a much larger intervention needed to address IPV at all socio-ecological levels.

## Data Availability

Deidentified data will be shared in Deep Blue for those wishing to conduct a secondary analysis of the data. Deep Blue is the University of Michigan’s permanent, safe, and accessible service for providing access to the work conducted by researchers at the University of Michigan. The repository has data access policies and procedures consistent with NIH data sharing policies. Submitted data will conform to relevant data standards. Data will be deposited within 1 year of completion of the funded project period for the award or upon acceptance of the results for publication. We will identify where the data will be available and how to access the data in any publications and presentations authored or coauthored regarding these data, as well as acknowledge the repository and funding source in any publications and presentations.
